# Co-payments for general practitioners in Denmark: an analysis using two policy models

**DOI:** 10.1186/s12913-016-1951-z

**Published:** 2017-01-05

**Authors:** Camilla Hansen, Despena Andrioti

**Affiliations:** 1 Centre for Evidence-Based Medicine, Odense University Hospital, University of Southern Denmark, Sdr. Boulevard 29, 5000 Odense, Denmark; 2 Centre of Maritime Health and Society (CMSS), University of Southern Denmark, Niels Borhs Vej 9, 6700 Esbjerg, Denmark

**Keywords:** Cost sharing, Out-of-pocket payments, General practice, Health Policy Triangle, Kingdon Model, Policy window

## Abstract

**Background:**

The increasing health expenditure for general practitioners (GPs) in Denmark requires that other ways of financing the health system are investigated. This study aims to analyse possibilities for implementing out-of-pocket payments to GPs in Denmark.

**Methods:**

The study was conducted as a literature review with 11 articles included. The Health Policy Triangle and the Kingdon Model were used in analysing and discussing the implementation of a cost-sharing policy with an emphasis on the out-of-pocket payments method.

**Results:**

The Danish Parliament has expressed mixed opinions about out-of-pocket payments, whereas the Danish population, the GPs and the media are against introducing payments. The public debate and the fact that Danes are used to healthcare being free of charge both work against introducing co-payments. However, experiences from Sweden, Norway and OECD countries serve to promote implementation, but at the expense of decreased accessibility for the most vulnerable population groups.

**Conclusions:**

Introducing out-of-pocket payments in Denmark may lead to decreased health expenditure, but also increased inequalities. Due to a lack of support from the relevant policy actors in the country, in addition to a lack of a policy window, it may not be possible to introduce out-of-pocket payments for GPs in Denmark in the short term.

## Background

In Denmark, general practitioners (GPs) have a gatekeeper function, where they are paid through a combination of fees-for-service and capitation, which are funded by the state, with the exception of vaccinations and health certificates [1]. The pressure on the Danish healthcare system has increased during recent decades. The Danish population is ageing and the proportion of elderly is increasing. Consequently, the GPs are treating patients with more complex diseases, which requires more resources [2].

The Danish healthcare systemt has an annual cost of approximately 94 billion DKK (€ 12.7 BN). Costs related to GPs represent 15% of the total annual cost, equal to about 14 billion DKK (€ 1.8 BN) [3]. Public health expenditure related to hospitals in Denmark has increased by 31% from 2007 to 2013. The expenditure for GPs has also risen by 15% in this period [4]. In 2006, the cost per capita connected to a GP was 700 DKK – in 2009, this cost increased by 20% to 840 DKK (from € 95 to € 112 respectively) [5].

Because of the increasing health expenditure connected to the use of GPs in Denmark, it is necessary to study other ways of financing. Due to an ageing Danish population, the demand for GPs may increase further in the future. Consequently, it has been speculated during recent years whether the introduction of out-of-pocket (OOP) payments to Danish GPs would be a solution to target the increasing expenditure. International literature has suggested that OOP payments to GPs reduce the costs by lowering the demand. For example, a study from the USA shows that co-payments can lower the annual cost by reducing demand, because when cost-sharing increases, the number of contacts with GPs decreases [6].

Currently, the level of OOP payments in Denmark is low compared to neighbouring countries, since only dental care, medication, physiotherapy and some psychological services are covered by co-payments [7]. To our knowledge, no studies have focused on the possibilities for implementing OOP payments in Denmark by applying the Health Policy Triangle and the Kingdon Model. On this basis, this study aims at analysing the possibilities for implementing the policy of OOP payments for GPs in Denmark by analysing relevant literature through the two mentioned policy models.

## Methods

### Data collection

This study was conducted as a literature review, which aimed at finding experiences with OOP payments. The search was based on a combination of the following search terms: out-of-pocket payment, co-payment, general practice, general practitioner, doctor, cost-sharing, demand, utilization of services. The search was conducted in three databases: PubMed Medline, EMBASE and Cochrane Library. The search included articles in English, Swedish, Norwegian and Danish, which have been published during the last ten years. Furthermore, the search was limited to studies from the Nordic countries, “Finland, Sweden, Denmark, Norway, and Iceland” [8], in order to make sure that the experiences were comparable to the Danish context. All searches were conducted during the last week of October 2014. In total, 1821 articles were found. The main inclusion criteria were that the articles discussed OOP payments for GPs and that they were from Nordic countries. The selection process resulted in five articles being included in the analysis.

Based on the chosen articles, a chain search was conducted, where articles that were possibly relevant were found from the list of references in the chosen articles. This made sure that a lot of relevant material was found and provided a quality control, since included articles had been approved by previous research [9, 10]. The same inclusion criteria were applied, and the chain search found six articles.

#### Media

The Danish database *Infomedia* was searched with the purpose of finding information on the media debate about OOP payments in Denmark. The following search terms were used: OOP payment [brugerbetaling], health care system [sundhedsvæsen], and general practitioner [praktiserende læge]. The search was limited to the past 12 months. Eight Danish newspapers were included in the search, which was conducted on 4^th^ November 2014 and gave 91 hits. In total, 14 articles from Danish newspapers were chosen for inclusion.

#### Electronic sources

The chosen articles were combined with information found on important websites. To gain knowledge on the healthcare systems, the websites of the National Boards of Health in Denmark, Norway, and Sweden were searched. Additionally, a website containing Danish laws, websites of two Danish GP organisations, the website of the Danish Parliament, and websites of eight Danish political parties were searched. Finally, the website www.cepos.dk was searched. CEPOS is an independent liberal organisation that aims at influencing the Danish political debate. CEPOS is in favour of OOP payments, which is why findings from this website are used with caution.

### Theoretical framework

The results were analysed using The Health Policy Triangle (HPT) and The Kingdon Model. The HPT is a theoretical framework that consists of four factors: *actors*, *content, context* and *process. Actors* refer to individuals or organisations who are either involved in the policy-making or affected by the policy. When defining possible actors, it is important to note what power and interest each actor possesses. *Content* refers to the actual content of the policy [11]. This is not applicable to this study, since the article is discussing the possible implementation of OOP payments and not the details about the payment itself. The *context* of a policy can be divided into four categories: *situational factors, structural factors, cultural factors,* and *international factors* [12]. *Situational factors* refer to situations in the surroundings, while *structural factors* describe the structure of the policy system. *Cultural factors* refer to how the surrounding culture can affect the policy, and *international factors* focus on how international conditions affect the policy. Finally, the *process* describes how the policy is initiated, developed, implemented and evaluated [11]. The latter two factors are outside the scope of this study, and will not be discussed further.

When discussing the potential implementation of OOP payments, the Kingdon Model is used. According to this, possible policies are likely to reach the agenda if a *policy window* is created. A policy window is created when three streams run together. The *problem stream* describes whether the general perception in the population is that the government should deal with the problem, and whether the government is able and willing to do so. The *policy stream* covers the debate in the media and among politicians, analysing the support of a solution. Finally, the *politics stream* describes whether the opinion among politicians is in favour or against the policy, which can be influenced by the national mood, campaigns or even replacement of the government [11].

## Results

### Actors

#### The Danish parliament

The Danish Parliament consists of 179 members and is responsible for making the laws in Denmark [13–15]. So the Danish Parliament has great direct power. The Parliament also decides on any reforms in the financing of the National Health Care System, including the possible introduction of OOP, and the majority of the Parliament members have to be in favour of this policy in order for it to be implemented. Since the General Election in 2011, the Parliament has consisted of eight Danish political parties - *Socialdemokratiet, Venstre, Dansk Folkeparti, Radikale Venstre, Konservative Folkeparti, Socialistisk Folkeparti, Enhedslisten* and *Liberal Alliance* - representing the entire political spectrum from left to right [16]. From 3^rd^ February 2014 to 28^th^ June 2015, the Danish government consisted of *Socialdemokratiet* and *Radikale Venstre* [17]. The government and the other parties on the left wing of the Parliament are against OOP payments to GPs. While *Socialdemokratiet, Socialistisk Folkeparti* and *Enhedslisten* completely reject introducing OOP payments to GPs, *Radikale Venstre* does not reject it completely [18–21]. In line with this, *Dansk Folkeparti* does not want to introduce OOP payments, because they fear it would lead to diseases being detected later [22]. On the rest of the right wing of the Parliament, *Venstre* and *Konservative Folkeparti* consider OOP payments a possibility, if used as an effort to reduce health expenditure [23, 24]. Finally, *Liberal Alliance* promotes OOP payments to GPs as a means to reduce taxes [25].

In general, the government is opposing OOP payments, whereas the opposition is more open-minded. This is in line with a literature review that has concluded that the political parties in Denmark change their opinion on OOP payments when they enter the government. For example, *Venstre* was against OOP payments until 2011, when the party became part of the government, while *Socialdemokratiet* was in favour. After the 2011 election, when a new government was introduced, the opinions changed so that *Venstre* was in favour of OOP payments, while *Socialdemokratiet* was against [26].

#### The Danish population

In the Danish political system, the population possesses an indirect power, because every Dane above the age of 18 is entitled to vote in political elections [15]. The attitude towards OOP payments among the population was analysed in 2011 by the Centre for Alternative Social Analysis (a non-profit organisation that produces research reports on aspects within the society) through questionnaires given to 1,200 Danish participants above the age of 15 years from all over the country. The study showed that Danes were against paying for GP visits. More specifically, 73% of the participants responded that there should be no payments for using GPs, because they pay a high amount of their income to taxes and thus they demand free access to health services when they need them. Only one percent believed that there should be cost-sharing practices introduced in the health system. Furthermore, 14% of the participants believed that there should be income-related co-payments to GPs in Denmark [27]. These findings are supported by other smaller scale studies in the following years [26].

#### The GPs

The GPs have the same power as the general population, because they can vote as private persons in political elections, assuming that they are Danish citizens. Furthermore, the GPs have two organisations: *Dansk Selskab for Almen medicin* and *Praktiserende Lægers Organisation*. These organisations can affect policy-making through scientific publications, through the media or through lobbying, which indicates an indirect power. The GPs are against introducing OOP payments. In a survey among 458 Danish GPs, 78% answered that they were against the general population having to pay for GP visits. Furthermore, the survey showed that 60% of the participants believed that payments would reduce both necessary and unnecessary consultations, mainly by the lower socio-economic population groups, possibly affecting their health [28].

#### The Danish media

The media is often called *The Fourth Estate* [29], because they have a major role in agenda setting [11]. The media thereby possesses great indirect power. This means that the media can keep the topic of OOP payments off the agenda if they are against introducing it, and thereby make it difficult for politicians to gain support from the general population. In general, the media present a picture that OOP payments are not a good idea. The majority of the articles in Danish newspapers are against OOP payments, on the grounds that these would increase inequality, reduce satisfaction from the utilization of services, with the long term effects not known, while diseases would be detected later, as access to GPs would be limited [30–38]. Despite the negative general picture, some stories promote paying for GPs, because they argue that payments can save a lot of money for Danish society and reduce taxes [39–43]. However, all articles found by this study that promote OOP payments are written either by CEPOS or by representatives of the Danish political party *Liberal Alliance*, who are in favour of OOP payments.

### The context of the policy

Denmark has faced a financial crisis since 2008, which represents the *situational factors*. This means that the unemployment rate has increased, causing reduced income among some Danes [44]. Because of this, the Danish population might be more resistant to co-payments, as they will further decrease their income.

The Danish Constitution has two rights that represent the *structural factors.* The first is freedom of speech, which allows the population to express any non-discriminating opinion [45], and the second right ensures that Danes can meet in groups [46]. Because of these factors, the public debate is potentially influencing politicians.

The *cultural factors* can be perceived as a barrier to the possible implementation of OOP payments policy. The Danish healthcare system has mainly been publicly funded since 1973, meaning that Danes are used to GP visits being free of charge. The fact that visits to the GPs have been publicly funded for so many years is generally seen as an obstacle for implementing co-payments, since the payment structure to GPs has an established history [26].

In contrast, *international factors* may promote implementation, since Danes may be influenced by neighbouring countries and international organisations. Co-payments to GPs have been an integral part of the health systems in Sweden and Norway since the establishment of the systems. Swedish citizens pay between 100 and 200 SEK (€ 10 and € 20) each time they visit a GP, with a yearly maximum of 1100 SEK (€ 115). Children and adolescents under the age of 20 do not pay for using GPs [47]. In Norway, citizens pay 127 DKK (€ 17) each time they visit a GP, with the exception of children below the age of 16 [48]. The yearly maximum is 1712 DKK (€ 230) [49]. The fact that Sweden and Norway have implemented co-payments for GPs might convince the Danish population that cost-sharing could be a good way to reduce public expenditure for health. Furthermore, a report published by OECD concluded that Denmark should consider introducing co-payments to GPs in an effort to lower public expenditure [50]. When international organisations publish studies in favour of OOP payments in Denmark, it gives the government some of the needed support, and thereby promotes implementation.

## Discussion

### Effects of introducing OOP payments for GPs in Denmark

The authors of a Danish literature review found that co-payments may lead to decreased demand, meaning that citizens will visit their GPs less often. More specifically, through a literature search in EconLit and a chain search, 51 quantitative studies published between January 1990 and December 2011 on the effects of OOP payments were included in the review. The authors highlighted that the vast majority of studies found that OOP payments reduced the number of services demanded, including the number of visits to GPs [51]. An economic analysis by CEPOS estimated that introducing a co-payment of 127 DKK (€ 17) per GP visit for every citizen above the age of 16, with an annual limit of 1,712 DKK (€ 230), would save society 2.3 billion DKK (€ 310 M), assuming a decreased demand of 10% [49]. Even though CEPOS is an organisation in favour of OOP payments, and this saving might be overestimated, the study suggested that OOP payments would reduce public health expenditure, which had also been observed in other countries that had introduced cost-sharing practices. The decrease is mainly observed in the use of pharmaceuticals and is supported by extensive literature, including a literature review that found that a fixed co-payment reduces drug use even when the co-payment is small [52].

OOP payments can lead to increased inequality [26]. A study based on the 2009 EU SILC survey showed that there is a link between co-payment and having unmet healthcare needs. 24.9% of the participants noted financial reasons as the main reason for their unmet medical needs. Furthermore, OOP payments would decrease accessibility to GPs, because payments would act as a barrier. According to a Norwegian study, the number of visits to GPs decreased when the accessibility decreased [53]. It can be assumed that the accessibility will decrease the most for the lowest socio-economic groups, because these groups have less money. This decreased accessibility is inconsistent with Danish health law, where it is said that there should be easy access to healthcare [54]. This discrepancy between Danish health law and OOP payments may hinder implementation. Moreover, a literature review showed that the vulnerable groups in society are more affected by OOP payments than the less vulnerable, richer groups [51]. A Danish nationwide survey established that immigrants and their descendants make lower use of services with OOP payments compared to ethnic Danes [55]. These studies showed that the lower socio-economic classes of Denmark might reduce their use of GPs the most, putting them at risk of ill health in the long term and creating inequalities. Increased inequality is a serious matter, because it can decrease overall life expectancy (LE) and can have economic consequences for society [56].

Even though LE in Sweden and Norway is higher than in Denmark [57], in the long term fewer visits to GPs may deteriorate health, as diseases will be diagnosed at a later and probably more costly stage, i.e. in hospital. To our knowledge, no studies have calculated the additional cost of treating patients at a more costly stage due to OOP payments to GPs in a Danish context. The fact that no studies exist may reflect the lack of support for OOP payments in Denmark.

For OOP payments to GPs to have no negative effects on health, it would require that the population is capable of distinguishing between necessary and unnecessary visits. A literature review on drug use and co-payment showed that payments for drugs decreased the use of both necessary and unnecessary drugs [58]. This study indicates that citizens might not be capable of making the right choice, due to the parameter of asymmetric information, thereby risking deterioration of their health in the long term because of OOP payments.

### Implementation of OOP payments in Denmark

The *problem stream* consists of the increasing health expenditure for GPs because of increased pressure on the healthcare system. In Denmark, it is the task of the government to make sure that public costs are kept within the approved limits [15], meaning that it is accepted by the population that the government has responsibility for dealing with the problem. However, since the *cultural factors* in the context of the policy have shown that Danes have been used to free access to GPs for decades, it might not be generally accepted that the government is entitled to introduce OOP payments.

Regarding the *policy stream,* the analysis of the media debate has shown that the media in general has been publishing stories against OOP payments. This means that even though OOP payments to GPs were generally accepted as a solution to decrease public expenditure on health, the support might be low. Furthermore, the analysis of the Danish population and GPs as stakeholders has shown that these actors are against introducing co-payments. This could mean that there is no *policy stream,* because the population and the media do not support OOP payments and can use the possibilities of expressing their opinion to influence the politicians. However, the analysis of the *international factors* has shown that the politicians can find support from Sweden, Norway, and the OECD.

The *politics stream* is represented by the analysis of the Danish Parliament. This analysis showed that the opinions among politicians differ, meaning that the government is against OOP payments, while the opposition is in favour. Since the general population is against OOP payments, the government might adjust its opinion accordingly in order to ensure re-election. However, as health expenditure continues to increase, the government might be forced to act against the opinion of the general population out of necessity. Furthermore, with the upcoming General Election, the government might change and a policy window may be created that could improve the chances of implementation. However, as shown in the analysis, there is a tendency for the opinions of the political parties to change, depending on whether they are a part of the government or not. If this tendency continues, future elections may not create such a policy window.

Overall, it can be argued that the *problem stream* and the *politics stream* run together, because OOP payments are generally accepted to be a means to decrease demand, and there is some support within the Parliament. However, all three streams are very unlikely to run together, because of the resistance towards OOP payments to GPs in the general population, the media, the GPs and parts of the Parliament. This means that it might be difficult to implement OOP payments in Denmark.

### Strengths and weaknesses

This study was a literature search, meaning that the strength of the analysis is dependent on the material found. The quality of the chosen literature was in general high, because all the chosen articles were found through scientific databases [10]. Additionally, the majority of the chosen articles have been published in leading scientific journals, meaning that the majority of them are high quality, peer-reviewed articles [59]. However, because our inclusion criteria only accepted studies from the Nordic countries, similar studies from outside the Nordic context were excluded. None of the included studies discussed the possibility that introducing OOP payments that are below the market value may in fact lead to an increased demand in some population groups. Studies discussing this may have been excluded because of the inclusion criteria. Consequently, this paper may overestimate the impact of OOP payments on the reduction in demand.

## Conclusions

This article aims to analyse the opportunities for potential implementation of OOP payments for GP visits in Denmark.

The results are based on the HPT and the Kingdon Model. By using the HPT, it becomes possible to simplify an otherwise complex political context, with the risk of missing important details not included in the model. Nevertheless, the use of the HPT and the Kingdon Model ensures that the most important factors are considered in the analysis of possibilities for implementing OOP payments in Denmark [11].

According to this analysis, four actors were identified as important for a potential implementation. The Danish Parliament possesses great power, but has opinions that differ over time. The general population and the GPs have a negative opinion towards OOP payments, while the Danish media mainly publishes negative stories on OOP payments. The contextual factors surrounding OOP payments to GPs were found to be both against and in favour of the policy. Overall, it may not be possible to implement OOP payments at the moment. The *problem stream, policy stream* and *politics stream* do not run together because of a lack of support from the general population, the GPs and the media, different attitudes in the Parliament, and a Danish culture that might work against introducing OOP payments.

## Abbreviations

BN: Billion
GP: General practitioner
HPT: The Health Policy Triangle
LE: Life expectancy
M: Million
OOP: Out-of-pocket
